# The importance of familial risk factors in children with ADHD: direct and indirect effects of family adversity, parental psychopathology and parenting practices on externalizing symptoms

**DOI:** 10.1186/s13034-022-00529-z

**Published:** 2022-12-02

**Authors:** Lea Teresa Jendreizik, Christopher Hautmann, Elena von Wirth, Christina Dose, Ann-Kathrin Thöne, Anne-Katrin Treier, Tobias Banaschewski, Katja Becker, Daniel Brandeis, Julia Geissler, Johannes Hebebrand, Sarah Hohmann, Martin Holtmann, Michael Huss, Thomas Jans, Anna Kaiser, Sabina Millenet, Luise Poustka, Priska Schneider, Manfred Döpfner

**Affiliations:** 1 School of Child and Adolescent Cognitive Behavior Therapy (AKiP), Faculty of Medicine and University Hospital Cologne, University of Cologne, Cologne, Germany; 2 Department of Child and Adolescent Psychiatry and Psychotherapy, Central Institute of Mental Health, Medical Faculty Mannheim, Heidelberg University, Mannheim, Germany; 3 Department of Child and Adolescent Psychiatry, Psychosomatics and Psychotherapy, Medical Faculty of the Philipps-University Marburg, Marburg, Germany; 4 Center for Mind, Brain and Behavior (CMBB), University of Marburg and Justus Liebig University Giessen, Marburg, Germany; 5 Department of Child and Adolescent Psychiatry and Psychotherapy, Psychiatric Hospital, University of Zürich, Zurich, Switzerland; 6 Neuroscience Center Zürich, University and ETH Zürich, Zurich, Switzerland; 7 Center of Mental Health, Department of Child and Adolescent Psychiatry, Psychosomatics and Psychotherapy, University Hospital of Würzburg, Würzburg, Germany; 8 Department of Child and Adolescent Psychiatry, Psychosomatics and Psychotherapy, University Hospital Essen, University of Duisburg-Essen, Essen, Germany; 9 LWL-University Hospital for Child and Adolescent Psychiatry, Ruhr-University Bochum, Hamm, Germany; 10 Department of Child and Adolescent Psychiatry and Psychotherapy, University Medical Center of the Johannes Gutenberg University Mainz, Mainz, Germany; 11 Department of Child and Adolescent Psychiatry and Psychotherapy, University Medical Center Göttingen, Göttingen, Germany; 12 Department of Child and Adolescent Psychiatry, Psychosomatics and Psychotherapy, University Hospital Tübingen, Tübingen, Germany; 13 Department of Child and Adolescent Psychiatry, Psychosomatics and Psychotherapy, Faculty of Medicine and University Hospital Cologne, University of Cologne, Cologne, Germany

**Keywords:** Attention-deficit/hyperactivity disorder, Oppositional defiant disorder, Family adversity, Parental mental health, Parenting, Structural equation modeling

## Abstract

**Background:**

Children experiencing unfavorable family circumstances have an increased risk of developing externalizing symptoms. The present study examines the direct, indirect and total effects of family adversity, parental psychopathology, and positive and negative parenting practices on symptoms of attention-deficit/hyperactivity disorder (ADHD) and oppositional defiant disorder (ODD) in children with ADHD.

**Methods:**

Data from 555 children (*M* = 8.9 years old, 80.5% boys) who participated in a multicenter study on the treatment of ADHD (ESCAschool) were analyzed using structural equation modeling (SEM).

**Results:**

The SEM analyses revealed that (a) family adversity and parental psychopathology are associated with both child ADHD and ODD symptoms while negative parenting practices are only related to child ODD symptoms; (b) family adversity is only indirectly associated with child ADHD and ODD symptoms, via parental psychopathology and negative parenting practices; (c) the detrimental effect of negative parenting practices on child ADHD and ODD symptoms is stronger in girls than in boys (multi-sample SEM); (d) there are no significant associations between positive parenting practices and child ADHD or ODD symptoms.

**Conclusions:**

Family adversity, parental psychopathology, and negative parenting practices should be routinely assessed by clinicians and considered in treatment planning.

Trial registration (18th December 2015): German Clinical Trials Register (DRKS) DRKS00008973.

**Supplementary Information:**

The online version contains supplementary material available at 10.1186/s13034-022-00529-z.

## Background

Externalizing disorders, including attention-deficit/hyperactivity disorder (ADHD) and oppositional defiant disorder (ODD), are among the most prevalent mental disorders in childhood and adolescence. ADHD is characterized by impairing and developmentally inappropriate levels of inattention, hyperactivity and impulsivity [1], and has an estimated worldwide prevalence of 3.4% in children and adolescents [45]. ODD is marked by irritable mood, defiant and disobedient behavior towards authority figures and vindictiveness [1], affecting about 3.6% of children and adolescents worldwide [45]. Both disorders are significantly more common in boys than in girls [16, 58]. Approximately half of children and adolescents diagnosed with ADHD are also affected by ODD [15, 33].

It is assumed that genetic and environmental risk factors accumulate to cause both of these externalizing disorders [2, 21]. The heritability of ADHD is estimated to be higher (about 74%) than that of ODD (about 61% [14, 22]). Most of the environmental risk factors that have been found to be associated with the onset of ADHD exert their influence during the prenatal and early postnatal period (e.g., exposure to toxins, extreme deprivation or traumatic brain injury early in life [10, 34, 54]. Environmental risk factors that exert their influence later in childhood and adolescence (e.g., socioeconomic status or parenting behaviors) have been found to be linked to the severity of ADHD symptoms and oppositional, aggressive, and nonsocial behaviors [9, 46, 50].

Back in 1975, Rutter and colleagues examined the associations between adverse family circumstances and psychological disorders in children and adolescents. They identified six family-related risk factors that were associated with the rate of child psychiatric disorders (i.e., severe marital discord, low social class, large family size, paternal criminality, maternal mental disorder, and foster placement) and revealed that the aggregate of these factors, rather than the presence of any single factor, was linked to psychopathology in the child [51]. Subsequently, Biederman et al. [4, 5] demonstrated that regardless of a child’s gender, the risk of developing ADHD and comorbid symptoms increased with an increasing number of family risk factors. Subsequent research yielded further support for a small but significant association between family adversity and child externalizing symptoms [36, 44].

Another field of environmental research focuses on the relationship between parental and child psychopathology, with studies reporting significant positive associations between child externalizing symptoms and parental symptoms of ADHD, depression, anxiety, and aggression [11, 13]. Besides genetic factors, several other processes that may be involved in the intergenerational transmission of psychopathology have been discussed [13, 23]. Among these, parenting behavior has been shown to be directly associated with child externalizing behaviors [43] and to mediate the association between parental psychopathology and child externalizing behaviors [3, 6, 24].

Bronfenbrenner's ecological systems theory [7] describes environmental factors which are relevant for child development, looking not only at the child and his or her immediate surroundings (microsystem) but also at larger systems of the child's environment (meso-, exo-, macro- and chronosystem). Based on this theory, the effects of family adversity (exosystem) on the child and his or her mental health can be thought to be mediated by familial factors that are more proximal to the child, such as parental psychopathology and parenting practices (microsystem). In line with this, the family stress model [12] postulates a theoretical process that links economic pressure in the family, via depressed parental mood and impaired parenting, to problematic adolescent adjustment. Several studies provided further evidence that family financial burden exacerbates child symptoms through increased depressive symptoms of the parents and a negative influence on parenting behavior [41, 47, 53, 56]. Extending the assumptions of the family stress model [12], we postulate that such an indirect effect is not specific to economic pressure in the family or depressive symptoms of parents. Rather, we hypothesize that both economic and psychosocial adversities in the family (family adversity) indirectly impact on child externalizing symptoms via parental psychopathology and parenting practices.

Previous studies examining possible moderating influences of child age and gender on the association between familial risk factors and externalizing symptoms in school-aged children yielded different findings depending on the particular familial risk factor investigated. While the associations of family adversity and parental psychopathology with child externalizing symptoms appear to be broadly independent of child age and gender [4, 11, 13, 36, 44], the association between parenting practices and child externalizing symptoms seems to vary as a function of child age and gender. According to a recent meta-analysis, parenting behaviors are more strongly related to child externalizing symptoms in older children than in younger children [43]. In addition, there is some evidence that girls may be more strongly influenced by negative parenting behaviors than boys (e.g., [27]).

To the best of our knowledge, the effects of family adversity, parental psychopathology, and parenting practices on child symptoms of ADHD and ODD have not yet been examined together within one comprehensive model, possibly because suitable methods for analysis require large sample sizes. The objectives of this study are to (a) determine direct, indirect, serial indirect and total effects of familial factors (i.e., family adversity, parental psychopathology, positive and negative parenting practices) on child ADHD and ODD symptoms and (b) investigate possible moderating effects of child age and gender in a large sample of children aged between 6 and 12 years with a diagnosis of ADHD.

## Methods

### Participants and procedure

This study used data drawn from the ESCAschool study (Evidence-based, Stepped Care of ADHD in school-aged children; [18]), a multicenter trial encompassing nine study sites in Germany (Cologne, Essen, Göttingen, Hamm, Mainz, Mannheim, Marburg, Tübingen, Würzburg). ESCAschool is part of the research consortium ESCAlife and investigated a stepped care approach for school-aged children with ADHD, involving individualized treatment strategies based on behavioral and pharmacological interventions. Participants were mainly recruited via the outpatient units of the participating study sites. The children included in ESCAschool (a) met the criteria for an ADHD diagnosis according to the *Diagnostic and Statistical Manual of Mental Disorders* (DSM, 5th ed.; [1]), (b) were between 6 and 12 years old, and (c) had an IQ score above 80. For the present study, we analyzed baseline data (i.e., before the start of the study treatment) of 555 children who were screened for the ESCAschool study and met all inclusion and exclusion criteria. More detailed information on the background, procedures, and inclusion and exclusion criteria for ESCAschool can be found in the published study protocol [18]. All parents and all children provided written informed consent to participate in the study. Ethics approval was provided by the local ethics committees for each participating center separately.

### Measures

The following measures were collected from clinicians and parents at the baseline assessment.

#### Family adversity (FAI)

Family adversity was measured using a modified version of the Family Adversity Index (FAI) originally developed by Rutter and colleagues [51]. The modified version, adapted from the German Mannheim Parent Interview [20], includes the following items: low parental education, crowded housing conditions, parental conflicts, parental delinquency, and parental mental disorder. Each item is coded dichotomously by a clinician (0 = *no*, 1 = *yes*) based on an interview with at least one parent. The five item scores are then summed together to form the index (value range: 0–5). Crowded housing conditions were defined as having less than one room per person. Parental conflicts were assumed in the case of single-parent families or if there were significant disputes between the parents. Parental delinquency was indicated if at least one parent had been sentenced to jail or penalized with a fine, or if a parent’s driving license had been revoked for at least 6 months. Finally, a parental mental disorder was coded if either parent had been diagnosed with a mental disorder during their lifetime.

#### Parental psychopathology (pPSYC)

Parental ADHD (pADHD) was measured using the German ADHD self-report questionnaire (ADHS-Selbstbeurteilungsbogen [ADHS-SB]; [48]), which was adapted to DSM-5 criteria for the purpose of the present study. Parents rated each of the 18 symptom items on a 4-point Likert scale ranging from 0 (*not present*) to 3 (*severe*), with higher scores indicating higher symptoms of parental inattention, impulsivity, and hyperactivity. The scores for all 18 items were summed together to form the total symptom score. In the present sample, the total symptom scale showed a high internal consistency (α = 0.91).

Parental symptoms of depression, anxiety and stress (pDAS) were assessed using the German short version (DASS-21; [40]) of the Depression Anxiety and Stress Scales (DASS; [37]). Parents rated each of the 21 items on a 4-point Likert scale ranging from 0 (*never*) to 3 (*very often*), with higher scores indicating a greater severity of parental symptoms. In the present study, a sum score was formed by considering all 21 items. In the present sample, the scale showed a high internal consistency (α = 0.91).

Parental aggression (pAGG) was assessed using the Aggression Questionnaire (AQ-12) by Bryant and Smith [8] in its German version [25]. The questionnaire consists of 12 items measuring physical aggression, verbal aggression, anger and animosity. Parents rated each item on a 6-point Likert scale ranging from 1 (*very atypical*) to 6 (*very typical*), with higher scores indicating more parental aggression. The scores on the 12 items were summed together to form the total symptom score. In the present sample, the total scale showed a good internal consistency (α = 0.86).

#### Parenting practices (pPAR, nPAR)

Positive parenting (pPAR) was measured using the German Questionnaire on Parenting Behavior (Fragebogen zum Erziehungsverhalten [FZEV]; [39]), which was developed on the basis of various English-language instruments (e.g., [55]). The scale consists of 13 items assessing positive, reinforcing and encouraging parenting behavior. Parents rated each item on a 4-point Likert scale ranging from 0 (*never*) to 3 (*very often*), with higher scores indicating a more frequent use of positive parenting practices. The scale value was formed by averaging the respective item scores. In the present sample, the scale demonstrated a good internal consistency (α = 0.85).

Negative parenting (nPAR) was measured using a short version of the Negative-Inept Parenting Scale (NIP) from the Assessment of Positive and Negative Parenting (FPNE; [30],, which was developed on the basis of the Management of Children’s Behavior Scale (MCBS, [42]). The scale used in the present study consists of 10 items, which measure inconsistent, impulsive and rigid parenting behavior. Parents rated each item on a 4-point Likert scale ranging from 0 (*never*) to 3 (*very often*), with higher scores indicating a more frequent use of negative parenting practices. The scale value was formed by averaging the respective item scores. In the present sample, the 10-item scale showed an acceptable internal consistency (α = 0.74).

#### Child ADHD and child ODD (cADHD, cODD)

Child symptoms of ADHD and ODD were each assessed independently by a clinician and by the parents. For the assessment of ADHD symptoms, the clinician used the 18 items of the German Diagnostic Checklist for ADHD (DCL-ADHS, DISYPS-III; [17], which reflect the criteria for ADHD according to the DSM-5 and the 10th edition of the *International Statistical Classification of Diseases and Related Health Problems* (10th ed.; [59]). For the assessment of oppositional symptoms, the clinician used the eight ODD items from the German Diagnostic Checklist for Oppositional Defiant and Conduct Disorder (CD) (DCL-SSV, DISYPS-III; [17]), which reflect the criteria for ODD according to the DSM-5 and ICD-10. The symptoms were explored using a German semi-structured clinical interview for ADHD, ODD and CD symptoms, which was conducted with at least one parent (ILF-EXTERNAL, DISYPS-ILF, [26]). Clinicians rated each item on a 4-point Likert scale ranging from 0 (*age-typical/not at all*) to 3 (*very much*, with higher scores indicating more pronounced child ADHD and ODD symptoms. The two scale values (ADHD, ODD were formed by averaging the respective item scores. In the present sample, the scales showed a good internal consistency (ADHD: α = 0.82; ODD: α = 0.83). Furthermore, a high interrater reliability has been reported, with an intraclass correlation of 0.91 (ADHD) and 0.94 (ODD) [57].

The parents assessed the children's ADHD and ODD symptoms using the German-language rating scales for ADHD (FBB-ADHS) and for ODD and CD (FBB-SSV, DISYPS-III; [17]), which are based on the DSM-5 and ICD-10. More specifically, parents rated 20 ADHD items (nine items on inattention, 11 items on hyperactivity) and eight ODD items on a 4-point Likert scale ranging from 0 (*not at all*) to 3 (*markedly*), with higher scores indicating more severe symptoms. Again, the two scale values (ADHD, ODD) were formed by averaging the respective item scores. In the present sample, the scales showed a good internal consistency, with a Cronbach’s alpha of 0.89 for each scale.

### Statistical analysis

In a first step, missing values, descriptive statistics and bivariate correlations were investigated. Analyses were performed using SPSS 27.0. To examine missing values, Little's (1988) missing completely at random (MCAR) test was performed. Key variables were examined for deviations from normality based on skewness and kurtosis. It was checked whether the intercorrelations of potential indicators of latent factors were positive and sufficiently strong (*r* ≥ 0.50) for the formation of latent factors. Child demographic variables (child age and gender) were tested for significant bivariate correlations with the familial variables (FAI, pADHD, pDAS, pAGG, pPAR, nPAR) and child symptoms (cADHD, cODD).

**Fig. 1:**
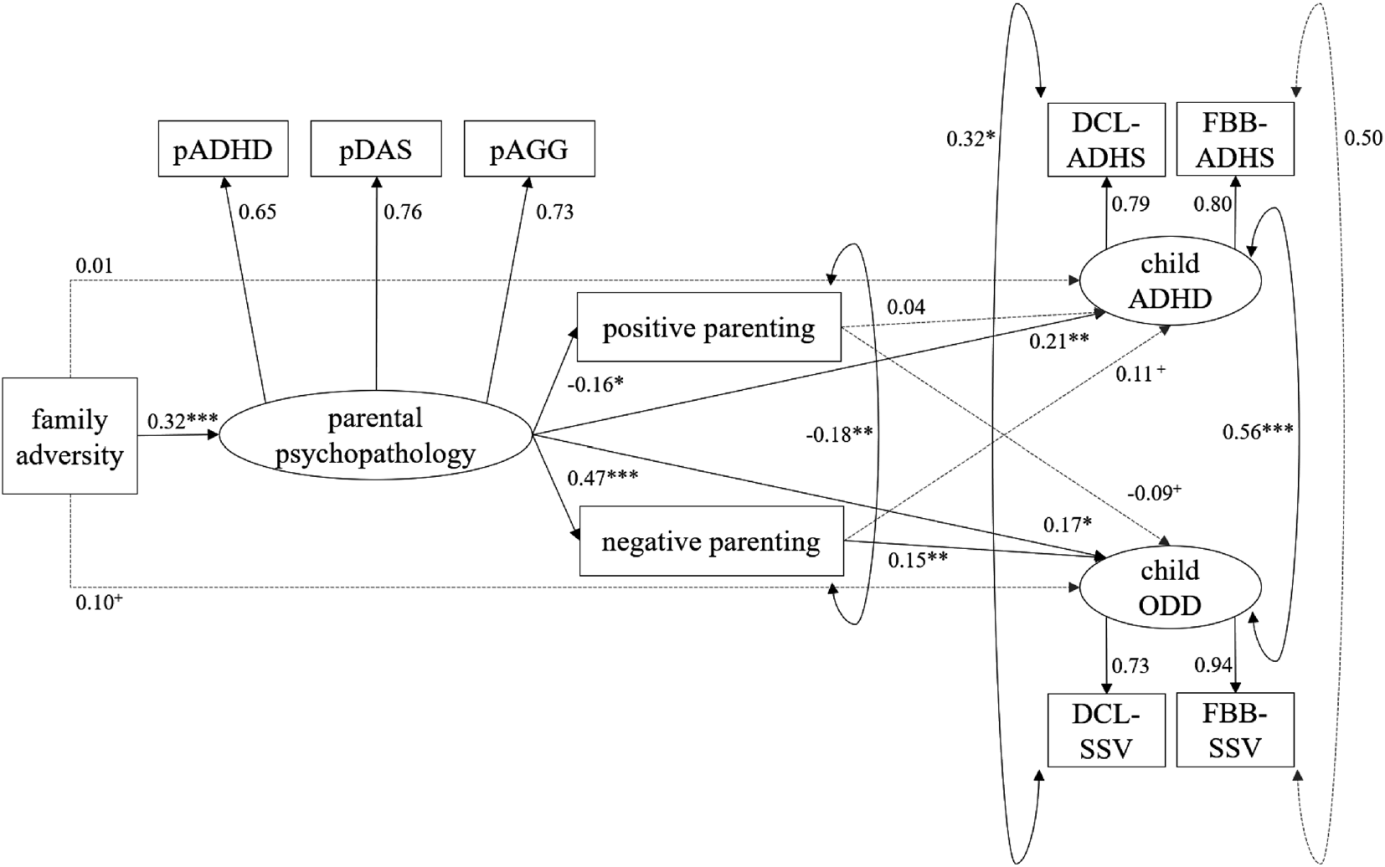
Structural equation model (SEM 2). Structural equation model depicting factor loadings, covariances and standardized path coefficients. Solid lines indicate significant paths, *p* < .05. The SEM 2 shown differs from SEM 1 by the added error covariance between positive and negative parenting practices. DCL-ADHS = clinician-rated child attention-deficit/hyperactivity disorder, DCL-SSV = clinician-rated child oppositional defiant disorder, FBB-ADHS = parent-rated child attention-deficit/hyperactivity disorder, FBB-SSV = parent-rated child oppositional defiant disorder, pADHD = parental attention-deficit/hyperactivity disorder (ADHS-SB), pAGG = parental aggression (AQ 12), pDAS = parental depression, anxiety and stress (DASS21). ^+^*p* < .10, *p < .05, **p < .01, ***p < .001

Within the main analyses, a confirmatory factor analysis (CFA) was performed and a structural equation model were tested: First, a *CFA* was conducted to assess the validity of the measurement models for the three latent factors parental psychopathology (pPSYC), child ADHD (cADHD), and child ODD (cODD). For the latent factor parental psychopathology (pPSYC), we used parental ADHD (pADHD), parental depression, anxiety and stress (pDAS), and parental aggression (pAGG) as indicators. For the two latent factors child ADHD (cADHD) and child ODD (cODD), corresponding clinician ratings (DCL-ADHS, DCL-SSV) and parent ratings (FBB-ADHS, FBB-SSV) were used as indicators and the error variances of the two indicators from one informant (clinician, parent) were allowed to covary. All three latent factors (pPSYC, cADHD, cODD) were allowed to covary. Second, an (*initial) structural equation model* (SEM 1) was checked for model fit. For SEM 1, we considered direct pathways from family adversity to parental psychopathology (FAI → pPSYC), from parental psychopathology to positive as well as negative parenting practices (pPSYC → pPAR/nPAR), and from all familial factors to child ADHD and child ODD (FAI/pPSYC/pPAR/nPAR → cADHD/cODD). Accordingly, the factors family adversity, parental psychopathology, (positive and negative) parenting practices, and child (ADHD and ODD) symptoms are arranged serially within SEM 1, and the positive and negative parenting practices and child ADHD and ODD symptoms are each arranged in parallel (see also Fig. 1). Consequently, SEM 1 enabled the determination of the direct effects of all familial factors on child ADHD and ODD symptoms (FAI/pPSYC/pPAR/nPAR → cADHD/cODD) as well as the indirect effects of family adversity (FAI) and parental psychopathology (pPSYC) on child ADHD and ODD symptoms (FAI → pPSYC → cADHD/cODD; pPSYC → pPAR/nPAR → cADHD/cODD), the serial indirect effects of family adversity (FAI) on child ADHD and ODD symptoms (FAI → pPSYC → pPAR/nPAR → cADHD/cODD), and the total effects of family adversity (FAI) and parental psychopathology (pPSYC) on child ADHD and ODD symptoms. Modification indices and theoretical considerations were used to examine reasonable adjustments to the SEM 1, and the model fit of the resulting model (i.e., SEM 2) was tested for its superiority over SEM 1.

*Multi-sample SEMs* were examined to reveal potentially moderating effects of (a) child age (split based on median age) and (b) gender. As a prerequisite, measurement invariance was tested beforehand. Configural invariance requires that the model configuration is identical in both groups (i.e., the same items belong to the same factors). Weak invariance additionally requires equal factor loadings in the groups and strong invariance additionally requires that the item intercepts are the same in the groups [35]. The total effects of family adversity and parental psychopathology on child symptoms and the direct effects of positive and negative parenting practices on child symptoms were determined for each of the two groups and compared using *χ*^*2*^ difference test.

Structural equation models were tested using the lavaan package (version 0.6–8; [49]) in R (version 4.1.0). For the SEM models, all variables were z-transformed and full information likelihood was used to handle missing values. As some variables were not normally distributed, a scaled test statistic was used (asymptotically equal to the Yuan-Bentler test statistic). Model fit was evaluated based on the comparative fix index (CFI), the standardized root mean square residual (SRMR), and the root mean square error of approximation (RMSEA). The model fit was considered acceptable if CFI ≥ 0.95, SRMR ≤ 0.08 and RMSEA ≤ 0.07 and good if CFI ≥ 0.95 and SRMR and RMSEA ≤ 0.05 [31, 35]. The χ^2^ test statistic was also inspected, although this index tends to increase along with the sample size and can therefore only be interpreted to a limited extent [35]. The direct, indirect, serial indirect and total effects of family adversity, parental psychopathology, and (positive and negative) parenting practices on child ADHD and ODD symptoms were determined, and bootstrapping with 1000 replications was used to obtain confidence intervals and standard errors of the estimated effects. Nested models were compared using the *χ*^*2*^ difference test, and non-nested models were compared using the Akaike information criterion (AIC) and the Bayesian information criterion (BIC).

## Results

### Study sample and descriptive statistics

The 555 children had a mean age of 8.9 years (*SD* = 1.5) and 80.5% were male. In total, 275 children (49.5%) had a combined type ADHD diagnosis, 222 children (40.0%) had a predominantly inattentive type ADHD diagnosis, and 58 children (10.5%) had a predominantly hyperactive-impulsive type ADHD diagnosis. About one-third (*n* = 189, 34.1 %) received medication for the treatment of ADHD. The following comorbid diagnoses were present in the study sample: ODD (*n* = 214, 38.6%), anxiety disorder (*n* = 41, 7.3%), CD (*n* = 37, 6.7%), tic disorder (*n* = 32, 5.8%), depressive disorder (*n* =18, 3.2%), and obsessive-compulsive disorder (*n* = 2, 0.4%). The participating parent was either the biological mother (87.2%), the biological father (7.4%), or another caregiver (5.4%). Little`s MCAR test was non-significant, which was in line with the assumption that the data were missing at random (*χ*^*2*^(217) = 232.94 *p* = 0.22). Descriptive statistics and bivariate correlations for the key and demographic variables are shown in Table 1.

**Table 1:** Descriptive Statistics and Bivariate Correlations for Key and Demographic Variables

	1. FAI	2. pADHD	3. pDAS	4. pAGG	5. pPAR	6. nPAR	7. cADHD (C)	8. cADHD (P)	9. cODD (C)	10. cODD (P)
1. FAI	1									
2. pADHD	0.20**	1								
3. pDAS	0.28**	0.50**	1							
4. pAGG	0.21**	0.48**	0.53**	1						
5. pPAR	0.03	− 0.04	− 0.13**	− 0.19**	1					
6. nPAR	0.11*	0.23**	0.36**	0.40**	− 0.23**	1				
7. cADHD (C)	0.06	0.17**	0.16**	0.12**	− 0.04	0.15**	1			
8. cADHD (P)	0.10*	0.16**	0.19**	0.10*	0.03	0.18**	0.63**	1		
9. cODD (C)	0.15**	0.14**	0.19**	0.13**	− 0.12**	0.20**	0.47**	0.33**	1	
10. cODD (P)	0.17**	0.16**	0.22**	0.16**	− 0.15**	0.25**	0.44**	0.54**	0.69**	1
Child gender (0 = b, 1 = g)	0.02	− 0.01	− 0.03	− 0.04	0.08	− 0.03	− 0.13**	− 0.07	− 0.11**	− 0.12**
Child age	− 0.05	− 0.07	− 0.05	− 0.02	− 0.14**	0.02	− 0.02	− 0.04	0.03	0.05
*n*	549	524	518	521	517	509	555	495	544	528
Missings in %	1.08	5.59	6.66	6.12	6.84	8.29	0	10.81	1.98	4.86
*M (SD)*	0.76 (0.85)	8.97 (8.58)	10.92 (8.44)	25.70 (9.22)	1.87 (0.38)	2.00 (0.39)	1.88 (0.47)	1.81 (0.53)	1.17 (0.65)	1.40 (0.71)
Min	0.00	0.00	0.00	12.00	0.77	1.10	0.72	0.20	0.00	0.00
Max	5.00	49.00	45.00	64.00	3.00	3.20	3.00	3.00	3.00	3.00
Skew	1.07	1.74	1.28	0.99	0.11	0.27	− 0.04	− 0.20	0.34	0.05
Kurtosis	1.21	3.52	1.89	1.22	− 0.19	0.09	− 0.70	− 0.48	− 0.28	− 0.74

b = boys, cADHD (C) = clinician-rated child attention-deficit/hyperactivity disorder (DCL-ADHS), cADHD (P) = parent-rated child attention-deficit/hyperactivity disorder (FBB-ADHS), cODD (C) = clinician-rated child oppositional defiant disorder (DCL-SSV), cODD (P) =  parent-rated child oppositional defiant disorder (FBB-SSV), FAI = Family Adversity Index, g = girls, nPAR = negative parenting (FPNE), pADHD = parental attention-deficit/hyperactivity disorder (ADHS-SB), pDAS = parental depression, anxiety and stress (DASS21), pPAR = positive parenting (FZEV)

^*^p < 0.05, **p < 0.01

### Model testing

The CFA resulted in a good model fit and confirmed the validity of the measurement models of the three latent factors parental psychopathology (pPSYC), child ADHD (cADHD), and child ODD (cODD, see Table 2). All factor loadings were of adequate strength and were significantly related to the respective latent factor (*β* > 0.68). We found a good model fit of the initial SEM (SEM 1) with direct pathways from family adversity to parental psychopathology, from parental psychopathology to positive as well as negative parenting practices, and from all familial factors to child ADHD and child ODD (see Table 2). Nevertheless, the modification indices (MI) suggested an extension of the model to include the error covariance between positive and negative parenting (MI > 10.00). Since this statistically based recommendation was also theoretically justifiable, the initial model was extended to include the suggested error covariance (SEM 2). As shown in Table 2, the superiority of the resulting model fit was confirmed by the result of the χ^2^ difference test. The coefficients of the postulated paths among the familial factors (FAI → pPSYC, pPSYC → pPAR/nPAR) each reached significance (see Fig. 1). In SEM 2, the explained variance (by all familial factors) in child ADHD was *R*^2^ = 7.5% and the explained variance in child ODD was *R*^2^ = 12.6%.

**Table 2:** Model fit parameters for CFA, SEM 1, SEM 2, and the alternative model

Model	*χ*^*2*^ (*df*)	*p*	CFI	SRMR	RMSEA	AIC	BIC	Δ*χ*^2^ (*df*)	*p*
CFA	5.21 (9)	0.82	1.00	0.01	< 0.001				
SEM 1	46.85 (24)	0.003	0.98	0.03	0.04				
SEM 2	32.51 (23)	0.09	0.99	0.02	0.03	13,570.21	13,751.60	14.16 (1)^a^	< 0.001
Alternative model	110.27 (23)	< 0.001	0.93	0.08	0.09	13,655.55	13,836.94		

In the CFA model the validity of the measurement models of the three latent factors parental psychopathology (pPSYC), child ADHD (cADHD), and child ODD (cODD) was assessed. In SEM 1, direct and indirect effects of family adversity (FAI), parental psychopathology (pPSYC), positive parenting (pPAR), and negative parenting (nPAR) on child ADHD and ODD symptoms were examined (FAI → pPSYC → pPAR/nPAR → cADHD/cODD). In SEM 2, SEM 1 was extended to include the error covariance of positive and negative parenting. The alternative model contained the following alternative arrangement of the familial factors, with otherwise unchanged paths: pPSYC → FAI → pPAR/nPAR → cADHD/cODD

AIC = Akaike information criterion, BIC = Bayesian information criterion, CFA = confirmatory factor analysis, CFI = comparative fix index, RMSEA = root mean square error of approximation, SRMR = standardized root mean square residual

^a^reference model = SEM 1

### Effects of familial factors on child externalizing symptoms

The direct, indirect and total effects of the four familial factors on child ADHD and ODD symptoms were determined in the extended SEM 2 model (see Table 3). First, considering the total effects of family adversity and parental psychopathology on *child ADHD symptoms*, as well as the direct effects of positive and negative parenting practices on child ADHD symptoms, only the total effect of parental psychopathology on child ADHD symptoms reached significance (*b* = 0.31, *SE* = 0.08, *β* = 0.25, *p* < 0.001). While the total effect of family adversity and the direct effect of negative parenting practices on child ADHD symptoms showed a trend for significance (FAI: *p* = 0.08; nPAR: *p* = 0.07), the direct effect of positive parenting practices did not. Second, considering the indirect and direct effects of family adversity and parental psychopathology on child ADHD symptoms, a significant indirect effect of family adversity on child ADHD symptoms via parental psychopathology was detected (*b* = 0.05, *SE* = 0.02, *β* = 0.07, *p* = 0.01), and a trend for a significant serial indirect effect of family adversity on child symptoms via parental psychopathology and negative parenting practices (*p* = 0.09). In contrast, the direct effect of family adversity on child ADHD symptoms was not significant. The direct effect of parental psychopathology on child ADHD symptoms was significant (*b* = 0.25, *SE* = 0.09, *β* = 0.21, *p* = 0.005) and the indirect effect of parental psychopathology on child ADHD symptoms via negative parenting practices showed a trend for significance (*p* = 0.09). Overall, an (exclusively indirect) effect of family adversity on child ADHD symptoms (FAI → pPSYC → cADHD) and a (direct) effect of parental psychopathology on child ADHD symptoms (pPSYC → cADHD) were revealed.

**Table 3:** Direct, Indirect and Total Effects of Familial Variables on Child Symptoms (SEM 2)

Effect	Path	*b* [95% CI]	*SE*	*β*	*p*
**Total**	**Family adversity (FAI) → child ADHD (cADHD)**	0.07 [− 0.01, 0.16]	0.04	0.09	0.08
Direct	FAI → cADHD ^b^	0.01 [− 0.08, 0.10]	0.04	0.01	0.85
Indirect	FAI → parental psychopathology (pPSYC) → cADHD	0.05 [0.01, 0.10]	0.02	0.07	0.01
Serial indirect	FAI → pPSYC → positive parenting (pPAR) → cADHD	− 0.00 [− 0.01, 0.00]	0.00	− 0.00	0.49
Serial indirect	FAI → pPSYC → negative parenting (nPAR) → cADHD	0.01 [− 0.00, 0.03]	0.01	0.02	0.09
**Total**	**Parental psychopathology (pPSYC) → child ADHD (cADHD)**	0.31 [0.15, 0.46]	0.08	0.25	< 0.001
Direct	pPSYC → cADHD	0.25 [0.07, 0.42]	0.09	0.21	0.005
Indirect	pPSYC → positive parenting (pPAR) → cADHD	− 0.01 [− 0.04, 0.02]	0.01	− 0.01	0.51
Indirect	pPSYC → negative parenting (nPAR) → cADHD	0.06 [− 0.01, 0.14]	0.04	0.05	0.09
**Direct**	**Positive parenting (pPAR) → child ADHD (cADHD) **^**a**^	0.04 [− 0.06, 0.11]	0.04	0.04	0.44
**Direct**	**Negative parenting (nPAR) → child ADHD (cADHD)**	0.09 [− 0.01, 0.18]	0.05	0.11	0.07
**Total**	**Family adversity (FAI) → child ODD (cODD)**	0.14 [0.06, 0.22]	0.04	0.19	0.001
Direct	FAI → cODD ^b^	0.08 [0.00, 0.17]	0.04	0.10	0.07
Indirect	FAI → parental psychopathology (pPSYC) → cODD	0.04 [0.01, 0.08]	0.02	0.06	0.04
Serial indirect	FAI → pPSYC → positive parenting (pPAR) → cODD	0.00 [0.00, 0.01]	0.00	0.01	0.18
Serial indirect	FAI → pPSYC → negative parenting (nPAR) → cODD	0.02 [0.01, 0.03]	0.01	0.02	0.005
**Total**	**Parental psychopathology (pPSYC) → child ODD (cODD)**	0.29 [0.15, 0.44]	0.07	0.26	< 0.001
Direct	pPSYC → cODD	0.20 [0.04, 0.35]	0.08	0.17	0.02
Indirect	pPSYC → positive parenting (pPAR) → cODD	0.02 [0.00, 0.05]	0.01	0.02	0.19
Indirect	pPSYC → negative parenting (nPAR) → cODD	0.08 [0.02, 0.15]	0.03	0.07	0.02
**Direct**	**Positive parenting (pPAR) → child ODD (cODD) **^**a**^	− 0.07 [− 0.16, 0.00]	0.04	− 0.09	0.09
**Direct**	**Negative parenting (nPAR) → child ODD (cODD)**	0.11 [0.03, 0.20]	0.04	0.15	0.008

Total and direct effects (bold text) of familial factors on child ADHD and child ODD symptoms were compared using χ^2^ difference test

^a^Corresponding effects differed significantly for child ADHD and child ODD based on χ2 difference test (*χ*^2^_*diff*_(1) = 7.23, *p* = 0.007)

Third, considering the total effects of family adversity and parental psychopathology, as well as the direct effects of positive and negative parenting practices on *child ODD symptoms*, the following three familial factors exerted a significant effect: family adversity (total effect: *b* = 0.14, *SE* = 0.04, *β* = 0.19, *p* = 0.001), parental psychopathology (total effect: *b* = 0.29, *SE* = 0.07, *β* = 0.26, *p* < 0.001), and negative parenting practices (direct effect: *b* = 0.11, *SE* = 0.04, *β* = 0.15, *p* = 0.008). The total effect of the fourth familial factor, positive parenting practices, only showed a trend for significance (*p* = 0.09). Fourth, we considered the indirect and direct effects of family adversity and parental psychopathology on child ODD symptoms. The analyses revealed a significant indirect effect of family adversity on child ODD symptoms via parental psychopathology (*b* = 0.04, *SE* = 0.02, *β* = 0.06, *p* = 0.04) and a serial indirect effect of family adversity on child ODD symptoms via parental psychopathology and negative parenting practices (*b* = 0.02, *SE* = 0.01, *β* = 0.02, *p* = 0.005). In contrast, the direct effect of family adversity on child ODD symptoms was not significant. Finally, parental psychopathology had both a direct effect on child ODD symptoms (*b* = 0.20, *SE* = 0.08, *β* = 0.17, *p* = 0.02) and an indirect effect on child ODD symptoms via negative parenting practices (*b* = 0.08, *SE* = 0.03, *β* = 0.07, *p* = 0.02). In summary, the analyses revealed an (exclusively indirect) effect of family adversity on child ODD symptoms (FAI → pPSYC → cODD; FAI → pPSYC → nPAR → cODD), a (direct and indirect) effect of parental psychopathology on child ODD symptoms (pPSYC → cODD; pPSYC → nPAR → cODD) and a (direct) effect of negative parenting practices on child ODD symptoms (nPAR → cODD).

When comparing the total (family adversity, parental psychopathology) or direct (positive and negative parenting practices) effects of the familial factors on child ADHD and child ODD symptoms (by comparing the model fits of the nested models with freely varying and equated path coefficients using χ2 difference test), only positive parenting practices had a significantly different effect on child ADHD and child ODD symptoms (*χ*^2^_diff_(1) = 7.23, *p* = 0.007).

An extension to model SEM 2, adding two additional pathways (FAI → pPAR/nPAR), provided the opportunity to examine even more potential indirect effects of the familial factors. However, the extended model did not provide a better model fit (*χ*^*2*^(21) = 27.95, *p* = 0.14, CFI = 1.00, SRMR = 0.02, RMSEA = 0.03; *χ*^*2*^_*diff*_(2) = 4.76, *p* = 0.09), and the total, direct, and indirect effects described above remained largely unchanged (for details see Additional file 1: Table A1).

### Moderating effects of child age and gender

#### Descriptive statistics and measurement invariance

Descriptive statistics and bivariate correlations are shown in the Additional file 1 (Tables A2 and A3) separately for younger and older children and for boys and girls. Configural as well as weak measurement invariance based on SEM 2 was shown across younger and older children but not across boys and girls. Specifically, for girls, the estimation of SEM 2 resulted in negative variances. Potential reasons for the estimation problems might have been the small sample size of girls (*n* = 108), the examination of a complex statistical model, and only two indicators for two of the three latent factors (cADHD, cODD) [35]. However, to nevertheless examine the moderating effect of gender, SEM 2 was simplified, and instead of the two latent factors (cADHD and cODD) with two indicators each (DCL-ADHS, FBB-ADHS; DCL-SSV, FBB-SSV), we calculated two separate SEMs with two manifest factors each. Specifically, one multi-sample SEM with clinician-rated child symptoms (DCL-ADHS, DCL-SSV) and one with parent-rated child symptoms (FBB-ADHS, FBB-SSV) were performed to examine the moderating influences of gender. As a result of the simplification of the model, configural and weak measurement invariance based on SEM 2 was shown across boys and girls (see Additional file 1: Table A4).

#### Effects of familial factors on child externalizing symptoms for younger and older children

For younger children only parental psychopathology (total effect: *b* = 0.36, *SE* = 0.10, *β* = 0.35, *p* < 0.001) and for older children none of the familial factors had a significant (total or direct) effect on *child ADHD symptoms*. However, for younger children family adversity showed a trend for a significant (total) effect (*p* < 0.10) and for older children negative parenting practices showed a trend for a significant (direct) effect on child ADHD symptoms (*p* = 0.06). The explained variance in child ADHD was *R*^2^ = 14.9% for younger children and *R*^2^ = 3.6% for older children. In both age groups, family adversity (younger children: *b* = 0.13, *SE* = 0.06, *β* = 0.19, *p* = 0.03; older children: *b* = 0.12, *SE* = 0.05, *β* = 0.17, *p* = 0.02) and parental psychopathology (younger children: *b* = 0.30, *SE* = 0.11, *β* = 0.30, *p* = 0.006; older children: *b* = 0.25, *SE* = 0.12, *β* = 0.21, *p* = 0.03) had significant (total) effects on *child ODD symptoms*. In addition, in both age groups negative parenting practices showed a trend for a significant (direct) effect on child ODD symptoms (younger children: *p* < 0.10; older children: *p* = 0.07). The explained variance in child ODD symptoms was *R*^2^ = 13.7% for younger children and *R*^2^ = 10.7% for older children. The direct effect of positive parenting did not reach significance in either age group or for either symptom domain (child ADHD, child ODD). None of the (total or direct) effects differed significantly between younger and older children. Further details are provided in Additional file 1 (Table A5).

#### Effects of familial factors on child externalizing symptoms for boys and girls

Due to the estimation problems of the SEM 2 in the group of girls and the calculation of two multi-sample SEMs for the moderator gender, separate estimates of direct and total effects resulted for the clinician rating and parent rating of child ADHD and ODD symptoms.

For boys, parental psychopathology (clinician rating: *b* = 0.33, *SE* = 0.08, *β* = 0.23, *p* < 0.001; parent rating: *b* = 0.32, *SE* = 0.10, *β* = 0.22, *p* = 0.001) and family adversity (only parent rating: *b* = 0.11, *SE* = 0.05, *β* = 0.11, *p* = 0.04) had a significant (total) effect on *child ADHD symptoms*. Additionally, negative parenting practices showed a trend for a significant (direct) effect on child ADHD symptoms for boys (only parent rating: *p* = 0.09). For girls, only negative parenting (only clinician rating: *b* = 0.33, *SE* = 0.14, *β* = 0.35, *p* = 0.02) had a significant (direct) effect on child ADHD symptoms. The explained variance in child ADHD symptoms was *R*^2^ = 5.0% (clinician rating) or *R*^2^ = 6.3% (parent rating) for boys and *R*^2^ = 9.7% (clinician rating) or *R*^2^ = 3.4% (parent rating) for girls. For boys, family adversity (clinician rating: *b* = 0.16, *SE* = 0.05, *β* = 0.16, *p* = 0.001; parent rating: *b* = 0.22, *SE* = 0.05, *β* = 0.21, *p* < 0.001) and parental psychopathology (clinician rating: *b* = 0.27, *SE* = 0.09, *β* = 0.19, *p* = 0.001; parent rating: *b* = 0.39, *SE* = 0.10, *β* = 0.27, *p* < 0.001) had a significant (total) effect on *child ODD symptoms*. Additionally, positive parenting practices showed a trend for a significant (direct) effect on child ODD symptoms for boys (clinician rating: *p* = 0.07; parent rating: p = 0.08). For girls, only negative parenting practices (clinician rating: *b* = 0.47, *SE* = 0.12, *β* = 0.52, *p* < 0.001; parent rating: *b* = 0.42, *SE* = 0.14, *β* = 0.45, *p* = 0.002) had a significant (direct) effect on child ODD symptoms. The explained variance in child ODD symptoms was *R*^2^ = 6.6% (clinician rating) or *R*^2^ = 12.4% (parent rating) for boys and *R*^2^ = 22.2% (clinician rating) or *R*^2^ = 15.7% (parent rating) for girls. The direct effects of negative parenting on child ADHD (only clinician rating) and child ODD (clinician and parent rating) showed significantly different path coefficients for boys and girls (by comparing the model fits of the nested models with freely varying and equated path coefficients using χ2 difference test). Further details are provided in Additional file 1 (Tables A6 and A7).

### Alternative arrangement of familial factors

To further test the plausibility of SEM 2, we examined an alternative arrangement of the familial factors. Specifically, instead of modeling a direct pathway from family adversity to parental psychopathology (FAI → pPSYC) and from parental psychopathology to (positive and negative) parenting practices (pPSYC → pPAR/nPAR), a direct pathway from parental psychopathology to family adversity (pPSYC → FAI) and from family adversity to (positive and negative) parenting practices (FAI → pPAR/nPAR) was provided within this alternative model. All other postulated pathways remained unchanged. As can be seen in Table 2, the model fit of the alternative model was not acceptable and both the AIC and the BIC suggested a superiority of the SEM 2 over the alternative model.

## Discussion

To the best of our knowledge, this is the first study to examine effects of family adversity, parental psychopathology, and parenting practices on ADHD and ODD symptoms together within one comprehensive model in a large sample of children with ADHD. The analyses performed supported a model inspired by Bronfenbrenner's ecological systems theory [7], in which the familial factors were ordered according to their proximity to the child. In the present study, family adversity was associated with a more pronounced parental psychopathology, which was in turn associated with more negative and fewer positive parenting practices. This finding is also in line with the assumptions of the family stress model [12]. More specifically, our results support the assumption that family adversity (e.g., low parental education, marital conflicts, parental delinquency, crowded housing conditions) is associated with increased psychopathological symptoms of the parents, which in turn have a negative impact on their parenting behavior. The strengths of the associations among these familial factors in the present study are comparable with previous study findings based on the family stress model [41, 47, 56].

### Effects of familial factors on child externalizing symptoms

Two of the four familial factors revealed significant effects on child ADHD symptoms: family adversity (indirect: FAI → pPSYC → cADHD) and parental psychopathology (total, direct). Three of the four familial factors revealed significant effects on child ODD symptoms: family adversity (total; indirect: FAI → pPSYC → cODD; serial indirect: FAI → pPSYC → nPAR → cODD), parental psychopathology (total; direct; indirect: pPSYC → nPAR → cODD), and negative parenting practices (direct). Accordingly, adverse family circumstances and psychopathological symptoms of parents were associated with more severe ADHD and ODD symptoms in children. In addition, inconsistent, impulsive, and rigid parenting behaviors (negative parenting practices) were related to more severe ODD symptoms in children. In contrast, positive, reinforcing and encouraging parenting behavior (positive parenting practices) was not associated with less severe externalizing symptoms in children. The effects of family adversity, parental psychopathology, and negative parenting practices on child externalizing symptoms were small and broadly in line with previous study findings [11, 13, 36, 43, 44]. The finding that negative parenting practices have more impact on children's externalizing behaviors than do positive parenting practices is also consistent with previous study findings [19, 28, 32, 43].

Neither ADHD symptoms nor ODD symptoms in children were directly related to family adversity. However, indirect effects of family adversity via parental psychopathology and serial indirect effects via parental psychopathology and negative parenting practices emerged. About 12.6% of the variance in child ODD and 7.5% of the variance in child ADHD symptoms was explained by the familial factors studied. The higher proportion of explained variance in child ODD symptoms compared to child ADHD symptoms in the presented SEM may be attributable to the fact that a greater number of the examined familial factors were associated with child ODD symptoms than with child ADHD symptoms. While it is necessary to take into account some statistical features in this regard (see: limitations and further studies), this finding is consistent with previous evidence suggesting less importance of genetic risk factors and a greater importance of environmental risk factors for ODD symptoms compared with ADHD symptoms [14].

### Moderating effects of child age and gender

The (total or direct) effects of the familial factors did not significantly differ between younger and older children, but did significantly differ between boys and girls. Inconsistent, impulsive and rigid parenting behaviors (negative parenting practices) were more strongly associated with child ADHD and ODD symptoms in girls than in boys. This finding is consistent with previous research (e.g., [27]), although conflicting evidence has also been reported [43]. More research is needed to clarify whether girls indeed show a greater sensitivity to negative parenting behaviors than do boys. Interestingly, the largest amount of explained variance in child symptoms was found for (clinician-rated) ODD symptoms in girls (*R*^*2*^ = 22.2%) and the smallest for (latent factor) ADHD symptoms in older children (*R*^*2*^ = 3.6%). Accordingly, it can be assumed that especially for ADHD symptoms in later childhood and adolescence, factors other than those studied here could be decisive for symptom severity.

### Limitations and recommendations for further studies

The findings of the present study should be interpreted in the context of several limitations. First, the data analyzed are cross-sectional. Unlike longitudinal data, cross-sectional data are not suitable for drawing conclusions about the direction of influence. However, an alternative model that changed the direction of the prediction of familial factors such that parental psychopathology preceded family adversity, parenting practices, and finally child externalizing symptoms resulted in an unacceptable model fit. Therefore, the reverse direction of influence can be considered unlikely. Nevertheless, some studies suggest a bidirectional rather than unidirectional relationship between child symptoms and family variables, especially parenting practices (e.g., [52]).

Second, the data collection was restricted to clinician and parent ratings of child symptoms. The consideration of several informants, such as clinicians, parents, teachers, and the child him/herself (from early adolescence), is central to a valid assessment of externalizing symptomatology in all relevant life domains. Future studies should additionally obtain a teacher's rating to provide as complete a picture as possible [38]. Although teacher ratings were requested and collected in the ESCAschool study, the number of available teacher ratings was considered too low (56%) for inclusion as a third indicator of child externalizing symptoms. Moreover, in view of evidence of a low correspondence between parental self-report and observational measures of parenting practices [29], in future studies, it would be valuable to include observations of parenting in order to rule out the suggestion that the purported associations between parental psychopathology and parenting practices may be purely attributable to same-informant effects.

Third, the Family Adversity Index, which is a tried and tested tool to assess adverse family circumstances [4], offers few concrete hints for deriving clinical implications. As the determination of individual risk factors may be more relevant to inform prevention and intervention approaches, future studies should examine individual risk factors (e.g., marital conflicts) instead of employing an index of family adversity. However, it should be critically noted that it may, in fact, be the combined presence of multiple, nonspecific, familial risk factors, rather than the presence of single, specific risk factors, that is associated with child symptom severity.

Fourth, the comparison of the impact of the familial factors on child ADHD and child ODD symptoms may be limited. Stronger associations between the investigated familial factors and child symptoms, and a correspondingly higher explained variance in child symptoms, were found for ODD symptoms than for ADHD symptoms in the present study. From a statistical perspective, it should be noted that all of the children had an ADHD diagnosis whereas only about 40% had an additional, comorbid ODD diagnosis. Moreover, in the present sample, the symptom expression was higher and the variance in symptoms was lower for ADHD symptoms than for the comorbid ODD symptoms. Therefore, it cannot be ruled out that the higher explained variance in the child symptoms for ODD than for ADHD was attributable to the smaller variances in ADHD symptoms.

Fifth, the findings on moderating effects by gender should be interpreted with caution. Even though the obvious estimation problems in the group of girls was circumvented by simplifying the SEM, the sample size must be considered to be small in relation to the complexity of the model studied [35]. Accordingly, the presented findings on moderating effects by gender should only be evaluated in terms of warranting further investigations.

## Summary and clinical implications

The present study provides evidence that (a) family adversity and parental psychopathology are associated with both child ADHD and ODD symptoms while negative parenting practices are only related to child ODD symptoms; (b) family adversity is only indirectly associated with child ADHD and ODD symptoms, via parental psychopathology and negative parenting practices; (c) the detrimental effect of negative parenting practices on child ADHD and ODD symptoms is stronger in girls than in boys; (d) there are no significant associations between positive parenting practices and child ADHD or ODD symptoms.

Understanding how familial factors are (directly and indirectly) related to child symptoms can inform the development and selection of effective interventions for children. Based on the present study, which provides evidence that children in adverse family circumstances and with psychologically impaired parents appear to be at increased risk for higher ADHD symptom severity and comorbid ODD symptoms, we recommend that these areas be routinely examined as part of the diagnostic process. In addition, to prevent ODD symptoms in children, and especially in girls, a detailed examination of parenting practices seems appropriate. Interventions addressing the parent–child interaction should presumably focus specifically on reducing negative parenting practices.

## Conclusions

Child development takes place in continuous interaction with the child’s direct (e.g., parent) and extended (e.g., familial, socioeconomic status) environment. It is important to consider that not only the direct parent–child interaction, but also more general environmental factors have a (sometimes indirect) impact on the child. For an etiological understanding of externalizing symptoms in children, especially ODD symptoms, clinicians should routinely consider familial factors such as adverse family circumstances, parental psychopathology, and (negative) parenting practices, and address them through appropriate interventions.

## Supplementary Information

**Additional file 1. **Importance of familial risk factors in ADHD_Table A1 to Table A7.

## Data Availability

The dataset can be obtained from the corresponding author upon reasonable request.
