# Supplementary material for: The importance of familial risk factors in children with ADHD: direct and indirect effects of family adversity, parental psychopathology and parenting practices on externalizing symptoms
Source: Child Adolesc Psychiatry Ment Health. 2022 Dec 2;16:96. doi: 10.1186/s13034-022-00529-z (PMC9717533; doi:10.1186/s13034-022-00529-z)
Supplement: Supplementary file 1 — Additional file 1. Importance of familial risk factors in ADHD_Table A1 to Table A7. [file 13034_2022_529_MOESM1_ESM.docx]

Table A1

Direct, Indirect and Total Effects for the Extension of Model SEM 2

| Effect | Path | *b* [95% CI] | *SE* | *ß* | *p* |
| --- | --- | --- | --- | --- | --- |
| **Total** | **Family adversity (FAI) → positive parenting (pPar)** | 0.03 [-0.07, 0.11] | 0.05 | 0.03 | 0.51 |
| Direct | FAI → pPar | 0.10 [-0.00, 0.18] | 0.05 | 0.10 | 0.04 |
| Indirect | FAI → parental psychopathology (pPSYC) → pPar | -0.07 [-0.11, -0.02] | 0.02 | -0.07 | 0.004 |
| **Total** | **Family adversity (FAI) → negative parenting (nPar)** | 0.11 [0.03, 0.20] | 0.05 | 0.11 | 0.01 |
| Direct | FAI → nPar | -0.05 [-0.14, 0.04] | 0.05 | -0.05 | 0.32 |
| Indirect | FAI → parental psychopathology (pPSYC) → nPar | 0.16 [0.101, 0.24] | 0.04 | 0.16 | < 0.001 |
| **Total** | **Family adversity (FAI) → child ADHD (cADHD)** | 0.07 [-0.01, 0.16] | 0.04 | 0.09 | 0.08 |
| Direct | FAI → cADHD | 0.01 [-0.08, 0.10] | 0.04 | 0.01 | 0.88 |
| Indirect | FAI → parental psychopathology (pPSYC) → cADHD | 0.05 [0.01, 0.10] | 0.02 | 0.07 | 0.02 |
| Indirect | FAI → positive parenting (pPAR) → cADHD | 0.00 [-0.01, 0.01] | 0.01 | 0.00 | 0.47 |
| Indirect | FAI → negative parenting (nPAR) → cADHD | -0.00 [-0.02, 0.01] | 0.01 | -0.01 | 0.40 |
| Serial indirect | FAI → pPSYC → pPAR → cADHD | -0.00 [-0.01, 0.00] | 0.00 | -0.00 | 0.48 |
| Serial indirect | FAI → pPSYC → nPAR → cADHD | 0.01 [-0.00, 0.03] | 0.01 | 0.02 | 0.09 |
| **Total** | **Parental psychopathology (pPSYC) → child ADHD (cADHD)** | 0.31 [0.15, 0.47] | 0.08 | 0.25 | < 0.001 |
| Direct | pPSYC → cADHD | 0.25 [0.07, 0.43] | 0.09 | 0.21 | 0.005 |
| Indirect | pPSYC → positive parenting (pPAR) → cADHD | -0.01 [-0.04, 0.02] | 0.02 | -0.01 | 0.49 |
| Indirect | pPSYC → negative parenting (nPAR) → cADHD | 0.07 [-0.01, 0.15] | 0.04 | 0.06 | 0.09 |
| **Direct** | **Positive parenting (pPAR) → child ADHD (cADHD)** | 0.04 [-0.06, 0.11] | 0.05 | 0.04 | 0.44 |
| **Direct** | **Negative parenting (nPAR) → child ADHD (cADHD)** | 0.09 [-0.01, 0.18] | 0.05 | 0.11 | 0.07 |
| **Total** | **Family adversity (FAI) → child ODD (cODD)** | 0.13 [0.05, 0.21] | 0.04 | 0.17 | 0.002 |
| Direct | FAI → cODD | 0.08 [-0.00, 0.17] | 0.04 | 0.10 | 0.08 |
| Indirect | FAI → parental psychopathology (pPSYC) → cODD | 0.04 [0.01, 0.08] | 0.02 | 0.06 | 0.04 |
| Indirect | FAI → positive parenting (pPAR) → cODD | -0.01 [-0.02, 0.00] | 0.01 | -0.01 | 0.23 |
| Indirect | FAI → negative parenting (nPAR) → cODD | -0.01 [-0.02, 0.01] | 0.01 | -0.01 | 0.38 |
| Serial indirect | FAI → pPSYC → pPAR → cODD | 0.00 [0.00, 0.01] | 0.00 | 0.01 | 0.17 |
| Serial indirect | FAI → pPSYC → nPAR → cODD | 0.02 [0.01, 0.04] | 0.01 | 0.02 | 0.005 |
| **Total** | **Parental psychopathology (pPSYC) → child ODD (cODD)** | 0.30 [0.15, 0.45] | 0.08 | 0.27 | < 0.001 |
| Direct | pPSYC → cODD | 0.20 [0.04, 0.35] | 0.08 | 0.17 | 0.02 |
| Indirect | pPSYC → positive parenting (pPAR) → cODD | 0.02 [0.00, 0.06] | 0.02 | 0.02 | 0.17 |
| Indirect | pPSYC → negative parenting (nPAR) → cODD | 0.08 [0.02, 0.16] | 0.04 | 0.08 | 0.02 |
| **Direct** | **Positive parenting (pPAR) → child ODD (cODD)** | -0.07 [-0.16, 0.00] | 0.04 | -0.09 | 0.09 |
| **Direct** | **Negative parenting (nPAR) → child ODD (cODD)** | 0.11 [0.03, 0.20] | 0.04 | 0.15 | 0.009 |

Table A2

Descriptive Statistics and Bivariate Correlations Separately for Younger and Older Children

|  | Younger children | | | Older children | | |  | Correlations | | | | | | | | | | | | |  |
| --- | --- | --- | --- | --- | --- | --- | --- | --- | --- | --- | --- | --- | --- | --- | --- | --- | --- | --- | --- | --- | --- |
|  | *n* | *M* | *SD* | *n* | *M* | *SD* | *t* | *1.* | *2.* | *3.* | *4.* | *5.* | | *6.* | | *7.* | *8.* | | *9.* | *10.* |  |
| *N* Boys (%) | 225 (80.6) | | | 222 (80.4) | | |  |  |  |  |  | |  |  |  | |  |  | |  | |
| 1. FAI | 277 | 0.78 | 0.86 | 272 | 0.75 | 0.84 | 0.36 | 1 | 0.20** | 0.27** | 0.16** | 0.03 | | 0.10 | | 0.11 | 0.15* | | 0.17** | 0.18** |  |
| 2. pADHD | 268 | 9.63 | 9.01 | 256 | 8.27 | 8.07 | 1.81 | 0.20** | 1 | 0.50** | 0.47** | -0.15* | | 0.21** | | 0.17** | 0.23** | | 0.17** | 0.17** |  |
| 3. pDAS | 265 | 11.28 | 8.86 | 253 | 10.53 | 7.97 | 1.01 | 0.29** | 0.50** | 1 | 0.59** | -0.15* | | 0.38** | | 0.22** | 0.31** | | 0.26** | 0.28** |  |
| 4. pAGG | 265 | 25.79 | 8.98 | 256 | 25.59 | 9.49 | 0.25 | 0.25** | 0.49** | 0.47** | 1 | -0.23** | | 0.45** | | 0.18** | 0.16* | | 0.17** | 0.15* |  |
| 5. pPAR | 268 | 1.92 | 0.39 | 249 | 1.82 | 0.37 | 2.96** | 0.03 | 0.08 | -0.11 | -0.15* | 1 | | -0.25** | | -0.06 | 0.10 | | -0.12 | -0.13 |  |
| 6. nPAR | 258 | 1.98 | 0.38 | 251 | 2.02 | 0.41 | -0.97 | 0.13* | 0.26** | 0.35** | 0.35** | -0.19** | | 1 | | 0.16** | 0.21** | | 0.21** | 0.25** |  |
| 7. cADHD (C) | 279 | 1.90 | 0.44 | 276 | 1.85 | 0.49 | 1.23 | 0.00 | 0.16** | 0.09 | 0.06 | -0.03 | | 0.15* | | 1 | 0.65** | | 0.47** | 0.43** |  |
| 8. cADHD (P) | 254 | 1.84 | 0.51 | 241 | 1.78 | 0.55 | 1.37 | 0.06 | 0.09 | 0.06 | 0.04 | -0.05 | | 0.17* | | 0.61** | 1 | | 0.28** | 0.45** |  |
| 9. cODD (C) | 276 | 1.14 | 0.63 | 268 | 1.19 | 0.67 | -0.78 | 0.14* | 0.12 | 0.11 | 0.10 | -0.12 | | 0.19** | | 0.48** | 0.39** | | 1 | 0.69** |  |
| 10. cODD (P) | 268 | 1.35 | 0.69 | 260 | 1.45 | 0.73 | -1.71 | 0.17** | 0.16* | 0.17** | 0.17** | -0.16* | | 0.25** | | 0.46** | 0.63** | | 0.68** | 1 |  |

*Notes.* Correlations for younger children (≤ 8.8 years) are presented above the diagonal and correlations for older children (> 8.8 years) below the diagonal. Age categories (younger vs. older children) were formed using a median split.

cADHD (C) = clinician-rated child attention-deficit/hyperactivity disorder (DCL-ADHS), cADHD (P) = parent-rated child attention-deficit/hyperactivity disorder (FBB-ADHS), cODD (C) = clinician-rated child oppositional defiant disorder (DCL-SSV), cODD (P) = parent-rated child oppositional defiant disorder (FBB-SSV), FAI = Family Adversity Index, nPAR = negative parenting (FPNE), pADHD = parental attention-deficit/hyperactivity disorder (ADHS-SB), pDAS = parental depression, anxiety and stress (DASS21), pPAR = positive parenting (FZEV).

* *p* < 0.05, ** *p* < 0.01.

Table A3

Descriptive Statistics and Correlations Separately for Boys and Girls

|  | Boys | | | Girls | | |  | Correlations | | | | | | | | |  |
| --- | --- | --- | --- | --- | --- | --- | --- | --- | --- | --- | --- | --- | --- | --- | --- | --- | --- |
|  | *n* | *M* | *SD* | *n* | *M* | *SD* | *t* | *1.* | *2.* | *3.* | *4.* | *5.* | *6.* | *7.* | *8.* | *9.* | *10.* |
| Child age | 447 | 8.95 | 1.49 | 108 | 8.89 | 1.35 | 0.38 |  |  |  |  |  |  |  |  |  |  |
| 1. FAI | 443 | 0.76 | 0.84 | 106 | 0.79 | 0.89 | -0.39 | 1 | 0.25** | 0.30** | 0.26** | 0.03 | 0.12* | 0.06 | 0.12* | 0.16** | 0.20** |
| 2. pADHD | 424 | 9.01 | 8.91 | 100 | 8.78 | 7.09 | 0.24 | -0.05 | 1 | 0.53** | 0.50** | -0.03 | 0.21** | 0.20** | 0.17** | 0.15** | 0.17** |
| 3. pDAS | 416 | 11.06 | 8.61 | 102 | 10.35 | 7.71 | 0.75 | 0.20* | 0.35** | 1 | 0.56** | -0.15** | 0.38** | 0.16** | 0.19** | 0.19** | 0.26** |
| 4. pAGG | 423 | 25.88 | 9.49 | 98 | 24.86 | 7.96 | 0.99 | -0.03 | 0.33** | 0.36** | 1 | -0.16** | 0.40** | 0.15** | 0.12* | 0.14** | 0.21** |
| 5. pPAR | 418 | 1.86 | 0.38 | 99 | 1.94 | 0.37 | -1.85 | 0.01 | -0.09 | 0.02 | -0.32** | 1 | -0.19** | -0.01 | 0.05 | -0.12* | -0.15** |
| 6. nPAR | 412 | 2.00 | 0.39 | 97 | 1.98 | 0.43 | 0.66 | 0.10 | 0.30** | 0.27** | 0.38** | -0.38** | 1 | 0.12* | 0.19** | 0.14** | 0.22** |
| 7. cADHD (C) | 447 | 1.91 | 0.46 | 108 | 1.76 | 0.48 | 3.09** | 0.07 | 0.02 | 0.12 | -0.09 | -0.12 | 0.27** | 1 | 0.61** | 0.45** | 0.43** |
| 8. cADHD (P) | 404 | 1.83 | 0.53 | 91 | 1.74 | 0.52 | 1.49 | 0.07 | 0.14 | 0.18 | -0.06 | 0.00 | 0.17 | 0.70** | 1 | 0.32** | 0.53** |
| 9. cODD (C) | 437 | 1.20 | 0.65 | 107 | 1.02 | 0.63 | 2.67** | 0.15 | 0.09 | 0.18 | 0.05 | -0.08 | 0.44** | 0.53** | 0.38** | 1 | 0.68** |
| 10. cODD (P) | 427 | 1.44 | 0.70 | 101 | 1.23 | 0.71 | 2.73** | 0.10 | 0.12 | 0.08 | -0.10 | -0.13 | 0.36** | 0.44** | 0.54** | 0.69** | 1 |

*Notes.* Correlations for boys are presented above the diagonal and correlations for girls below the diagonal.

cADHD (C) = clinician-rated child attention-deficit/hyperactivity disorder (DCL-ADHS), cADHD (P) = parent-rated child attention-deficit/hyperactivity disorder (FBB-ADHS), cODD (C) = clinician-rated child oppositional defiant disorder (DCL-SSV), cODD (P) = parent-rated child oppositional defiant disorder (FBB-SSV), FAI = Family Adversity Index, nPAR = negative parenting (FPNE), pADHD = parental attention-deficit/hyperactivity disorder (ADHS-SB), pDAS = parental depression, anxiety and stress (DASS21), pPAR = positive parenting (FZEV).

* *p* < 0.05, ** *p* < 0.01.

Table A4

Model Fit Parameters of the Multi-Sample SEMs for Younger and Older Children and for Boys and Girls

| Model | χ^2^ (df) | *p* | CFI | SRMR | RMSEA | Δ *χ^2^* (df) | *p* |
| --- | --- | --- | --- | --- | --- | --- | --- |
| ‘Age’ |  |  |  |  |  |  |  |
| Configural invariance | 73.11 (46) | .007 | 0.981 | 0.033 | 0.046 |  |  |
| Weak invariance | 72.26 (50) | .021 | 0.984 | 0.034 | 0.041 | 1.24 (4) | 0.87 |
| Strong invariance | 86.97 (56) | .005 | 0.978 | 0.040 | 0.046 | 14.96 (6) | 0.02 |
| ‘Gender’ (C) |  |  |  |  |  |  |  |
| Configural invariance | 49.09 (24) | .002 | 0.967 | 0.033 | 0.060 |  |  |
| Weak invariance | 46.98 (26) | .007 | 0.970 | 0.033 | 0.054 | 0.56 (2) | 0.75 |
| Strong invariance | 63.85 (32) | .001 | 0.957 | 0.041 | 0.059 | 18.08 (6) | < 0.01 |
| ‘Gender’ (P) |  |  |  |  |  |  |  |
| Configural invariance | 48.95 (24) | .002 | 0.968 | 0.034 | 0.060 |  |  |
| Weak invariance | 46.60 (26) | .008 | 0.972 | 0.034 | 0.055 | 0.64 (2) | 0.72 |
| Strong invariance | 58.69 (32) | .003 | 0.964 | 0.039 | 0.055 | 12.27 (6) | 0.06 |

*Notes.* Configural and weak measurement invariance based on SEM 2 was confirmed for younger and older children, but estimation problems (i.e., negative variances) occurred for boys and girls. Consequently, SEM 2 was simplified, and instead of the two latent factors with two indicators, two separate SEMs with two manifest factors each were calculated. Specifically, one multi-sample SEM with clinician-rated child symptoms (DCL-ADHS, DCL-SSV) and one with parent-rated child symptoms (FBB-ADHS, FBB-SSV) were analyzed under the assumption of weak measurement invariance.

(C) = clinician-rated child symptoms, (P) = parent-rated child symptoms.

CFI = comparative fix index, RMSEA = root mean square error of approximation, SRMR = standardized root mean square residual.

Table A5

Direct, Indirect and Total Effects on Child ADHD and Child ODD Symptoms in the Multi-Sample SEM for Younger and Older Children

|  |  | Younger children (*n* = 279) | | | | Older children (*n* = 276) | | | |
| --- | --- | --- | --- | --- | --- | --- | --- | --- | --- |
| Effect | Path | *b* [95% CI] | *SE* | *ß* | *p* | *b* [95% CI] | *SE* | *ß* | *p* |
| Total | FAI → cADHD | 0.01 [-0.01, 0.22] | 0.06 | 0.13 | < 0.10 | 0.03 [-0.08, 0.13] | 0.05 | 0.04 | 0.53 |
| Total | pPSYC → cADHD | 0.36 [0.16, 0.54] | 0.10 | 0.35 | < 0.001 | 0.13 [-0.11, 0.44] | 0.14 | 0.11 | 0.33 |
| Direct | pPar → cADHD | 0.09 [-0.08, 0.19] | 0.07 | 0.13 | 0.16 | -0.02 [-0.12, 0.10] | 0.06 | -0.03 | 0.72 |
| Direct | nPAR → cADHD | 0.06 [-0.09, 0.17] | 0.07 | 0.08 | 0.38 | 0.12 [-0.01, 0.25] | 0.07 | 0.17 | 0.06 |
| *R^2^* (cADHD) |  | 14.9 % | | | | 3.6 % | | | |
| Total | FAI → cODD | 0.13 [0.02, 0.25] | 0.06 | 0.19 | 0.03 | 0.12 [0.02, 0.24] | 0.05 | 0.17 | 0.02 |
| Total | pPSYC → cODD | 0.30 [0.08, 0.52] | 0.11 | 0.30 | 0.006 | 0.25 [0.04, 0.51] | 0.12 | 0.21 | 0.03 |
| Direct | pPAR → cODD | -0.02 [-0.13, 0.06] | 0.05 | -0.03 | 0.69 | -0.09 [-0.19, 0.01] | 0.05 | -0.12 | 0.11 |
| Direct | nPAR → cODD | 0.10 [-0.02, 0.21] | 0.06 | 0.14 | < 0.10 | 0.11 [0.00, 0.23] | 0.06 | 0.16 | 0.07 |
| *R^2^* (cODD) |  | 13.7 % | | | | 10.7 % | | | |

*Notes.* Total and direct effects of familial factors on child symptoms for younger and older children were compared using χ^2^ difference tests, and no effect differed significantly (all *p* > 0.05) for younger and older children.

cADHD = child attention-deficit/hyperactivity disorder, cODD = child oppositional defiant disorder, FAI = Family Adversity Index, nPar = negative parenting, pPAR = positive parenting, pPSYC = parental psychopathology.

Table A6

Direct, Indirect and Total Effects on Clinically Rated Child ADHD and Child ODD Symptoms in the Multi-Sample SEM for Boys and Girls

|  | | Boys (*n* = 447) | | | | | Girls (*n*= 108) | | | |
| --- | --- | --- | --- | --- | --- | --- | --- | --- | --- | --- |
| Effect | Path | *b* [95% CI] | *SE* | *ß* | *p* | *b* [95% CI] | | *SE* | *ß* | *p* |
| Total | FAI → cADHD | 0.05 [-0.04, 0.15] | 0.05 | 0.05 | 0.25 | 0.06 [-0.13, 0.31] | | 0.11 | 0.06 | 0.59 |
| Total | pPSYC → cADHD | 0.33 [0.18, 0.50] | 0.08 | 0.23 | < 0.001 | 0.03 [-0.56, 0.76] | | 0.36 | 0.01 | 0.93 |
| Direct | pPar → cADHD | 0.03 [-0.07, 0.13] | 0.05 | 0.03 | 0.54 | -0.03 [-0.26, 0.12] | | 0.12 | -0.03 | 0.79 |
| Direct | nPAR → cADHD ^a^ | 0.02 [-0.11, 0.13] | 0.06 | 0.02 | 0.77 | 0.33 [0.08, 0.65] | | 0.14 | 0.35 | 0.02 |
| *R^2^* (cADHD) |  | 5.0 % | | | | 9.7 % | | | | |
| Total | FAI → cODD | 0.16 [0.07, 0.26] | 0.05 | 0.16 | 0.001 | 0.12 [-0.02, 0.32] | | 0.09 | 0.12 | 0.18 |
| Total | pPSYC → cODD | 0.27 [0.11, 0.43] | 0.09 | 0.19 | 0.001 | 0.34 [-0.32, 1.07] | | 0.37 | 0.16 | 0.36 |
| Direct | pPAR → cODD | -0.09 [-0.20, 0.01] | 0.05 | -0.09 | 0.07 | 0.08 [-0.12, 0.29] | | 0.10 | 0.08 | 0.77 |
| Direct | nPAR → cODD ^a^ | 0.04 [-0.08, 0.16] | 0.06 | 0.04 | 0.57 | 0.47 [0.23, 0.70] | | 0.12 | 0.52 | < 0.001 |
| *R^2^* (cODD) |  | 6.6 % | | | | 22.2 % | | | | |

*Notes.* Total and direct effects of familial factors on child symptoms for boys and girls were compared using χ^2^ difference tests.

^a^ Corresponding effects differed significantly based on χ^2^ difference test for boys and girls (cADHD: χ^2^*_diff_* (1) = 5.032, *p* = 0.02; cODD: χ^2^*_diff_* (1) = 64.947, *p* < 0.001).

cADHD = child attention-deficit/hyperactivity disorder, cODD = child oppositional defiant disorder, FAI = Family Adversity Index, nPar = negative parenting, pPAR = positive parenting, pPSYC = parental psychopathology.

Table A7

Direct, Indirect and Total Effects on Parent-Rated Child ADHD and Child ODD Symptoms in the Multi-Sample SEM for Boys and Girls

|  | | Boys (*n* = 447) | | | | | Girls (*n* = 107) | | | |
| --- | --- | --- | --- | --- | --- | --- | --- | --- | --- | --- |
| Effect | Path | *b* [95% CI] | *SE* | *ß* | *p* | *b* [95% CI] | | *SE* | *ß* | *p* |
| Total | FAI → cADHD | 0.11 [0.00, 0.21] | 0.05 | 0.11 | 0.04 | 0.08 [-0.09, 0.32] | | 0.10 | 0.09 | 0.41 |
| Total | pPSYC → cADHD | 0.32 [0.12, 0.51] | 0.10 | 0.22 | 0.001 | 0.23 [-0.29, 1.02] | | 0.34 | 0.11 | 0.49 |
| Direct | pPar → cADHD | 0.07 [-0.04, 0.17] | 0.05 | 0.07 | 0.21 | 0.04 [-0.20, 0.29] | | 0.12 | 0.04 | 0.72 |
| Direct | nPAR → cADHD | 0.10 [-0.02, 0.22] | 0.06 | 0.10 | 0.09 | 0.14 [-0.12, 0.41] | | 0.15 | 0.15 | 0.36 |
| *R^2^* (cADHD) |  | 6.3 % | | | | 3.4 % | | | | |
| Total | FAI → cODD | 0.22 [0.13, 0.32] | 0.05 | 0.21 | < 0.001 | 0.07 [-0.11, 0.33] | | 0.13 | 0.08 | 0.56 |
| Total | pPSYC → cODD | 0.39 [0.20, 0.62] | 0.10 | 0.27 | < 0.001 | 0.09 [-0.57, 0.74] | | 0.33 | 0.04 | 0.78 |
| Direct | pPAR → cODD | -0.09 [-0.18, 0.01] | 0.05 | -0.09 | 0.08 | -0.01 [-0.22, 0.21] | | 0.11 | -0.01 | 0.90 |
| Direct | nPAR → cODD ^a^ | 0.09 [-0.02, 0.22] | 0.06 | 0.09 | 0.11 | 0.42 [0.00, 0.18] | | 0.14 | 0.45 | 0.02 |
| *R^2^* (cODD) |  | 12.4 % | | | | 15.7 % | | | | |

*Notes.* Total and direct effects of familial factors on child symptoms for boys and girls were compared using χ^2^ difference tests.

^a^ Corresponding effect differed significantly based on χ^2^ difference test for boys and girls (χ^2^*_diff_* (1) = 6.695, *p* = 0.01).

cADHD = child attention-deficit/hyperactivity disorder, cODD = child oppositional defiant disorder, FAI = Family Adversity Index, nPar = negative parenting, pPAR = positive parenting, pPSYC = parental psychopathology.
